# Near-complete telomere-to-telomere *de novo* genome assembly in Egyptian clover (*Trifolium alexandrinum*)

**DOI:** 10.1093/dnares/dsae036

**Published:** 2024-12-18

**Authors:** Mitsuhiko P Sato, Ramadan A Arafa, Mohamed T Rakha, Amero A Emeran, Sachiko Isobe, Kenta Shirasawa

**Affiliations:** Kazusa DNA Research Institute, Chiba 292-0818, Japan; Plant Pathology Research Institute, Agricultural Research Center, Giza 12619, Egypt; Faculty of Agriculture, Kafrelsheikh University, Kafr El-Sheikh 33516, Egypt; Faculty of Agriculture, Kafrelsheikh University, Kafr El-Sheikh 33516, Egypt; Kazusa DNA Research Institute, Chiba 292-0818, Japan; Kazusa DNA Research Institute, Chiba 292-0818, Japan

**Keywords:** clover, genome assembly, long-read technology, telomere-to-telomere

## Abstract

Egyptian clover (*Trifolium alexandrinum* L.), also known as berseem clover, is an important forage crop to semi-arid conditions that was domesticated in ancient Egypt in 5,5000 BCE and introduced and well adapted to numerous countries including India, Pakistan, Turkey, and Mediterranean region. Despite its agricultural importance, genomic research on Egyptian clover has been limited to developing efficient modern breeding programs. In the present study, we constructed near-complete telomere-to-telomere-level genome assemblies for 2 Egyptian clover cultivars, Helaly and Fahl. Initial assemblies were established by using highly fidelity long-read technology. To extend sequence contiguity, we developed a gap-targeted sequencing (GAP-Seq) method, in which contig ends are targeted for sequencing to obtain long reads bridging 2 contigs. The total length of the resultant chromosome-level assemblies was 547.7 Mb for Helaly and 536.3 Mb for Fahl. These differences in sequence length can be attributed to the expansion of DNA transposons. Population genomic analysis using single-nucleotide polymorphisms revealed genomic regions highly differentiated between 2 cultivars and increased genetic uniformity within each cultivar. Gene ontologies associated with metabolic and biosynthetic processes and developmental processes were enriched in these genomic regions, indicating that these genes may determine the unique characteristics of each cultivar. Comprehensive genomic resources can provide valuable insights into genetic improvements in Egyptian clover and legume genomics.

## Introduction

Forage crops are essential for livestock production. Clover, which encompasses a range of *Trifolium* species, is an important forage crop.^1^ The genus *Trifolium* is a member of the cool-season annual legume family and is genetically similar to the genus *Medicago*.^2,3^ Among *Trifolium*, red clover (*T. pratense*), and white clover (*T. repens*) are popular species for forage production worldwide.^4,5^ Subterranean clover (*T. subterraneum*) is resilient to poor-quality soil where other clovers cannot survive.^6^

Egyptian clover (*T. alexandrinum*), or berseem clover, originated in the eastern Mediterranean region and was domesticated in ancient Egypt since 5,500 BCE.^7^ Egyptian clover was introduced to numerous countries during the late 19th and early 20th centuries.^7^ The primary targets of Egyptian clover breeding programs are high biomass production, ease of cultivation, and substantial nitrogen fixation capacity.^7^ Since this species is allogamous in nature, like red and white clovers, a mass selection method is usually applied in the breeding programs, resulting in the maintenance of genetic diversity within a cultivar to avoid inbreeding depression by self-crossings.^8^ Genomics is a promising tool for accelerating breeding programs by playing a crucial role in the rapid development of new varieties and ensuring resilience and adaptability in changing climates.^9^ Although genome information on red clover, white clover, and subterranean clover is publicly available,^10–15^ no genome data for Egyptian clover have been reported thus far, except for the chromosome number of 2*n* = 2*x* = 16.^16^

Advances in high-throughput sequencing technologies, such as the use of ultra-long read sequences, co-barcoding of long DNA fragments, Hi–C, and optical mapping methods, have accelerated the determination of high-quality novel genome sequences at telomere-to-telomere (T2T) levels.^17,18^ Several plant genomes have been sequenced at the T2T level, including those with large genome sizes or tetraploid structures.^19^ However, the establishment of T2T- or chromosome-level genome assemblies using advanced technologies is not always possible. A combination of long reads and target sequencing would be useful for extending sequence contiguity.

Genomic information is required to facilitate breeding programs for Egyptian clovers with complex genetic backgrounds. The widely cultivated Egyptian clover cultivars in Egypt belong to the ‘Meskawi’ botanical group (multicut type), which can regrow after being cut and thus is capable of delivering multiple cuts during the growing season. Being a basal-branching type, ‘Meskawi’ is characterized by the aggregation of buds in the basal crown area, which is left after cutting to allow for regrowth.^20^ Helaly cultivar, which belongs to Meskawi realized depending on forage production vigour. On the other hand, single-cut ‘Fahl’ Egyptian clover is another botanical group of clover that lacks regrowth ability. As a stem-branching type, buds are vertically distributed along the main stem and, thus, removed by taking the first and only cut from the crop.^21^ In this study, we presented the near-complete T2T-level genome sequences for 2 cultivars, Helaly for the multicut type and Fahl for single-cut type. Genetic diversity within and between cultivars was evaluated on the basis of genome sequence data. These findings provide genetic and genomics context for Egyptian clover, paving the way for future breeding programs for Egyptian clover and other related species.

## Materials and methods

### Plant materials

Two *T. alexandrinum* cultivars, Helaly and Fahl, were used in this study. Both cultivars were obtained from the Agricultural Research Center, Giza, Egypt. The Fahl cultivar is commonly used for single cuts as it has poor regeneration ability. However, Helaly cultivar is commonly used in the north of Egypt and can give 5 to 6 cuts of fodder, and has good regeneration ability after cutting.^22^

### De novo genome sequencing and assembly

Leaf samples were collected from a single plant of each cultivar and subjected to high-molecular-weight genomic DNA extraction using a Genomic-tip 500/G (Qiagen). Short-read sequence data were obtained to estimate the genome sizes of the Helaly and Fahl cultivars. The genome sequence libraries were constructed with Swift 2S Turbo Flexible DNA Library Kit (Swift Biosciences, Michigan, United States) and sequenced on the DNBSEQ-G400 (MGI Tech, Shenzhen, China). Generated short read sequences were processed by trimming low-quality bases (quality value < 10) and adapter sequences (AGATCGGAAGAGC) using PRINSEQ and fastx_clipper from the FASTX-Toolkit, respectively. The genome sizes of both cultivars were estimated using *k*-mer distribution analysis (*k* = 21) with Jellyfish software (v.2.3.0).^23^

For *de novo* genome sequence assembly, high-molecular-weight genomic DNAs were sheared with a g-TUBE (Covaris, Massachusetts, United States) by centrifugation at 1,600 × *g*, and long-read sequencing libraries were prepared using the SMRTbell Express Template Prep Kit v2 with a barcoded overhang adapter kit (PacBio, California, United States) for multiplexing. The resulting libraries were separated using BluePippin (Sage Science, Massachusetts, United States) to remove short DNA fragments (<20 kb). Sequence data was obtained using the Sequel II system (PacBio), demultiplexed, and converted into HiFi reads using SMRT Link pipeline (PacBio). The HiFi reads were assembled in contig sequences using Hifiasm version 0.19.5,^24^ in which the parameter that removed 20 bases from both ends of the reads was set.

**Fig. 1.**
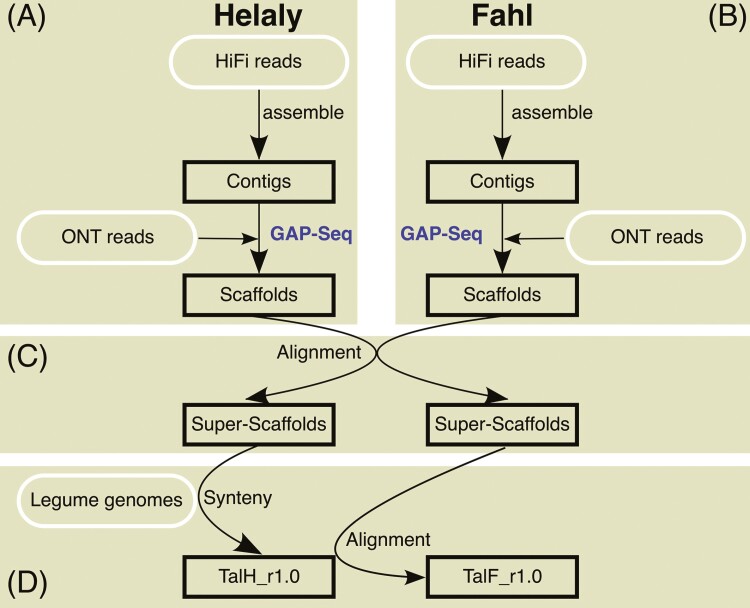
Assembly scheme of *T. alexandrinum*. The workflows and the data used for the analysis are shown in (A) for Halely and (B) for Fahl. The subsequent data analytic flows are shown in (C and D).

The obtained contig sequences were assembled into pseudomolecule sequences using a strategy involving another type of long-read sequence and alignment between the cultivars and among closely related species (Fig. 1). To link the contigs with another long-read sequencing technology, libraries were prepared using the Ligation Sequencing Kit V14 (Oxford Nanopore Technologies, Oxford, United Kingdom) and sequenced using a flow cell (R10.4.1) with GridION (Oxford Nanopore Technologies). Subsequently, the adaptive sampling mode was employed. In Helaly, the target sequences were simply set at 5,000 bp, at both ends of the HiFi contigs. In Fahl, on the other hand, in accordance with the results of Helaly that repeat sequences at contig ends might disrupt the exact enrichment of the target sequences, targets were set at the first 5,000 bp, with less than 50% of repeat regions within 100,000 bp at the end of the contigs. Base calling was performed using the super-accuracy model using MinKNOW. Low-quality reads (quality value < 10) and short reads (less than 20 kb) were trimmed using Chopper software.^25^ Long reads were used to scaffold the HiFi contigs using the LINKS^26^ software. The parameters influencing scaffolding contiguity and accuracy were tested as follows: *k*-mer length (15, 21, 31, 41, and 51-mer), a minimum number of links (5, 10, and 15), a distance between *k*-mer pairs (4,000 to 40,000), allowable error distance (2%, 5%, and 10%), and maximum link ratio between the 2 best contig pairs (0.1 and 0.3). All other parameters were set to their default values. The accuracy of the parameters was evaluated based on large discrepancies in the genome structure between cultivars using D-genies.^27^

To create super-scaffolds, scaffold sequences of the 2 cultivars were then aligned with each other using minimap2 (v2.24)^28^ and visualized using D-genies.^27^ Based on this alignment, if 1 scaffold sequence from 1 cultivar bridged 2 scaffolds from the other cultivar, the latter 2 scaffolds were manually connected with 100 Ns to construct super-scaffolds.

Finally, based on synteny and collinearity in the legume genomes, chromosome-level sequences were generated by aligning the super-scaffolds with the chromosome sequences of the closely related species *T. repens*,^13^*Medicago truncatula*,^29^ and *Pisum sativum*^30^ using MCScanX.^31^ The results were visualized using SynVisio.^32^ For the allotetraploid *T. repens*, because 2 subgenomes, probably from *T. occidentale* and *T. pallescens*, exhibit almost identical structures, only the subgenome of *T. occidentale* was used. The completeness of the genome sequences was evaluated using BUSCO v5.5.0 with embryophyte_odb10 data,^33^ LTR Assembly Index (LAI),^34^ telomere_finder (https://github.com/MitsuhikoP/telomere_finder),^19^ and tidk (https://github.com/tolkit/telomeric-identifier).

### Gene prediction and annotation

Potential protein-coding genes were predicted using Helixer version 0.3.1,^35^ which is a prediction tool for *de novo* gene models that combines Deep Learning and a Hidden Markov Model. To validate the results of gene prediction using Helixer, full-length cDNA sequence data were obtained using the PacBio Iso-Seq method. Total RNAs were isolated from the leaves, flowers, stems, and roots of each cultivar using the Plant Total RNA Mini Kit for Woody Plant (FAVORGEN, Taiwan) and used for library preparation according to the protocol of the ISO-Seq Express Template Preparation for Sequel and Sequel II System (PacBio). Libraries were sequenced using the Sequel II system (PacBio). Transcript isoforms for each sample were generated using the Iso-Seq analysis pipeline (PacBio) implemented in SMRT Link (PacBio). Functional annotations of predicted genes were assigned using eggNOG-Mapper 2.1.8^36^ against the eggNOG 5.0^37^ database. Gene clustering was performed using OrthoFinder,^38^ in which the gene sequences were from *Arabidopsis thaliana*,^39^*Glycine max*,^40^*Lotus japonicus*,^41^ and *M. truncatula*^29^ were involved.

Repetitive sequences were detected with RepeatMasker v4.1.6 (https://www.repeatmasker.org) using the repeat sequences obtained from the pseudomolecule sequences using RepearModeler v2.0.5 (https://www.repeatmasker.org) and from a dataset registered in Repbase.^42^

### Population genomic analysis

Genetic variations within each cultivar were investigated by double-digest restriction site-associated DNA sequencing (ddRAD-Seq) technique.^43^ Genomic DNA was extracted from 144 Helaly seedlings using the sbeadex DNA extraction kit on the oKtopure system (LGC, United Kingdom) and from 182 Fahl ovules of seeds by the method of Dellaporta et al.^44^ The extracted DNA was used to construct ddRAD-Seq libraries with PstI and MspI enzymes.^45^ ddRAD-Seq reads were obtained using DNBSEQ-G400 (MGI Tech) and trimmed using the same methods of short-read sequencing. Potential contaminated reads from archaea, bacteria, viral, plasmid, human, and vector sequences were investigated using Kraken 2.^46^ The cleaned reads were mapped onto Helaly pseudomolecule sequences as a reference using Bowtie2,^47^ and sequence variants were called using BCFtools.^48^ High-confidence biallelic single-nucleotide polymorphisms (SNPs) were selected using VCFtools^49^ (parameters: minDP, 5; minQ, 200; maf, 0.05; max-maf, 0.95; and max-missing, 0.8). Tajima’s *D* and Weir and Cockerman’s *F*_*ST*_ using a sliding window approach along pseudomolecule sequences was computed using VCFTools with a nonoverlapping 100-kb window size.^49–51^ On the basis of the Tajima’s *D* and *F*_*ST*_ values, genomic regions highly differentiated between 2 cultivars and increased genetic uniformity within each cultivar were detected as significant at *p* < 0.01 determined by 10,000 permutations.

Enrichment analyses for gene ontology (GO) were performed using topGO in the R package,^52^ and Fisher’s exact test and multiple corrections were performed with a false discovery rate.

## Results

### Genome sequence and scaffolding with GAP-Seq

On the basis of *k*-mer frequency analysis using whole genome sequences with short reads (99 Gb for Helaly and 105 Gb for Fahl), the estimated genome sizes of *T. alexandrinum* were 486 Mb for Helaly and 474 Mb for Fahl (Fig. S1). As the peaks of *k*-mer multiplicity, 125 for Helaly and 143 for Fahl were used. The presence of 2 peaks in the *k*-mer distribution suggested a high level of genomic heterogeneity in both varieties.

**Fig. 2.**
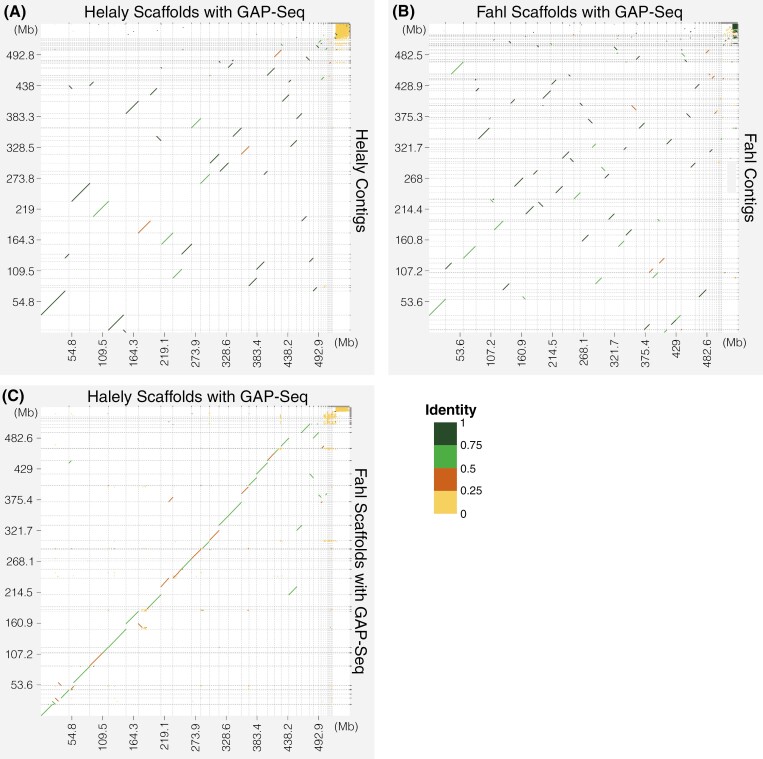
Contiguity improvement among assemblies at each step of the workflow. (A) Comparison between contigs and scaffolds using GAP-Seq in Helaly. (B) Comparison between contigs and scaffolds using GAP-Seq in Fahl. (C) Comparison of scaffolds between Helaly and Fahl. Horizontal and vertical dotted lines indicate the ranges of contigs or scaffolds. Line colours represent sequence identity.

For genome assembly of Helaly, we acquired 1.48 million HiFi reads, totalling 32.0 Gb (genome coverage of 65.8X), and assembled them into 514 contigs spanning 548 Mb with an N50 length of 14.6 Mb (Fig. 1A, Table 1). To efficiently scaffold the contigs, we employed a novel gap-targeted sequencing (GAP-Seq) strategy to target contig ends with a total length of 5.14 Mb (0.94% of the assembly size) for sequencing. Target sequencing with the Oxford nanopore technology (ONT) adaptive sampling strategy allowed sequencing of 7.69 Gb consisting of 3.35 million reads. Of these long reads, 1.28 million reads were recognized as target sequences, and 56,513 reads with sufficient accuracy (>10 QV score) and length (>20 kb) were selected as high-quality reads. More than half of the high-quality reads were omitted because of sequence stretching in the direction proximal to the target regions. Comparison of the scaffolding contiguity and accuracy among multiple parameter sets of the scaffolding software revealed that the distance between *k*-mer pairs affected the scaffolding efficiency, whereas other parameters had a small impact. To avoid mis-assembly, more conservative parameters than defaults were employed as follows: *k*-mer of 51; at least 15 links; distance between *k*-mer pairs, 20,000 bp; allowable error on this distance, 2%; and maximum link ratio between 2 best contig pairs, 0.1. As a result, 61 contigs were connected scaffolds to the ONT long reads and generated 482 scaffolds. The number of resultant sequences was 482, and the contig N50 length was extended to 21.3 Mb (Fig. 2A).

**Table 1.** Statistics of the genome assembly and gene prediction at each step of the workflow.

	Contig	Scaffold	Super-scaffold	Final assembly	Pseudomolecule
**Helaly**					
Amount of input reads (base)	32,047,672,472	7,686,104,399	–	–	–
Total length (bp)	547,551,778	547,720,126	547,721,526	547,722,026	504,434,753
Number of contigs	514	482	468	463	8
N50 length (bp)	14,593,844	21,275,908	49,702,867	55,867,553	55,867,553
Complete genome BUSCO	–	–	–	99.00%	99.00%
Number of genes	–	–	–	48,862	39,945
Complete protein BUSCO	–	–	–	98.90%	98.80%
**Fahl**					
Amount of input reads (base)	16,894,207,360	10,852,944,769	–	–	–
Total length (bp)	536,093,576	536,248,754	536,250,254	536,250,754	495,879,160
Number of contigs	316	260	245	240	8
N50 length (bp)	8,564,604	22,132,424	47,517,876	56,147,078	56,147,078
Complete genome BUSCO	–	–	–	99.00%	98.80%
Number of genes	–	–	–	42,966	39,585
Complete protein BUSCO	–	–	–	99.10%	98.90%

Consistent with the procedures performed for Helaly, we implemented the same strategy for Fahl. We acquired 0.81 million HiFi reads, totalling 16.9 Gb (genome coverage of 35.6X), and assembled them into 316 contigs spanning 536 Mb with an N50 length of 8.56 Mb (Fig. 1B and Table 1). Using the GAP-Seq strategy for 915-kb (0.17%) target regions, 10.9 Gb consisting of 8.28 million ONT long reads were obtained. Of these ONT reads, 829 thousand reads were recognized as target sequences and 72,353 reads had sufficient accuracy (>10 QV score) and length (>20 kb). With the high-quality ONT reads, 79 contigs were connected and generated 260 scaffolds. The N50 length was extended to 22.1 Mb (Fig. 2B).

### Constructing near-telomere-to-telomere genome assemblies

The scaffolds were manually integrated into super-scaffolds based on the alignments between the Helaly and Fahl cultivars (Fig. 1C and 2C). In total, 482 Helaly scaffolds and 260 Fahl scaffolds were complementary aligned to obtain 13 super-scaffolds each for Helaly and Fahl, all of which were longer than 10 Mb. Other short scaffolds that were not aligned were retained as unplaced scaffolds of the *T. alexandrinum* genome sequences. The N50 lengths improved to 49.7 Mb and 47.5 Mb for Halely and Fahl, respectively.

**Fig. 3.**
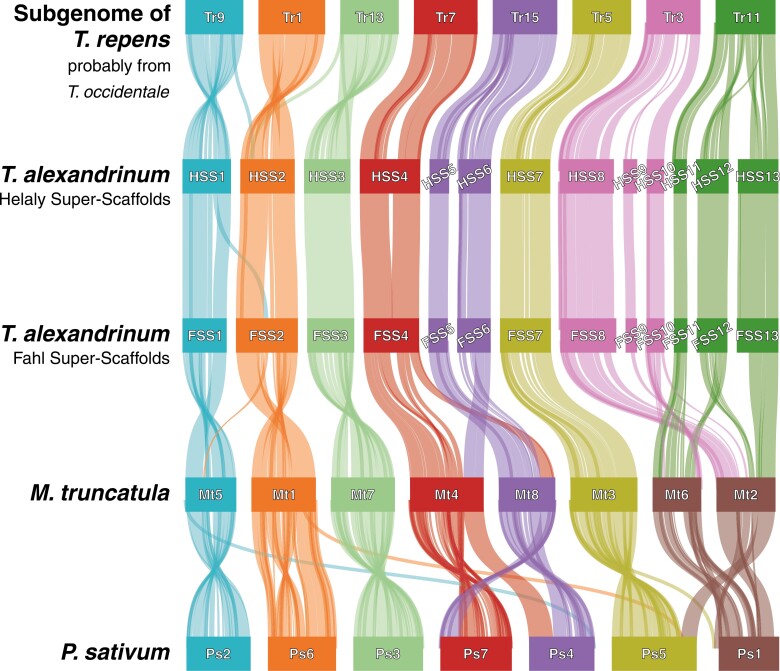
Synteny and collinearity of genes in *T. repens*, *T. alexandrinum* (Halely, super-scaffold), *M. truncatula*, and *P. sativum*. A subgenome, probable *T. occidentale* was used to represent *T. repens*.

To construct chromosome-level assemblies, the 13 super-scaffolds of Helaly were aligned with a subgenome of *T. occidentale* haplotype sequence of *T. repens*, based on synteny and collinearity (Figs. 1D and Fig. 3). Five super-scaffolds were consistent with the 5 chromosomes of the *T. occidentale* haplotype sequence. The other 8 super-scaffolds were aligned to the remaining 3 *T. occidentale* chromosomes to generate 3 pseudomolecule sequences. These processes yielded 8 pseudomolecule sequences corresponding to the basic number of *T. alexandrinum* chromosomes. Subsequently, the 13 super-scaffolds of Fahl were aligned with Helaly, in which 5 super-scaffolds corresponded to 5 chromosomes and the remaining 8 super-scaffolds were connected to 3 pseudomolecule sequences. In both varieties, the resultant assemblies, which included both the pseudomolecule sequences and the retained short scaffolds, were named TalH_r1.0 for Helaly (547.7 Mb in total length) and TalF_r1.0 for Fahl (536.3 Mb). The nomenclature and direction of the pseudomolecule sequences were based on the 8 chromosomes of the *T. occidentale* haplotype sequence of the *T. repens* genome.

The quality of pseudomolecule sequences was assessed using 3 methods. In both TalH_r1.0 and TalF_r1.0, telomere repeats were identified at all ends of the 8 pseudomolecule sequences (Fig. S2). The complete BUSCO score of TalH_r1.0 was 99.0%, and the single-copy and duplicated BUSCO scores were 94.5% and 4.5%, respectively. The complete BUSCO score of TalF_r1.0 also was 99.0%, and the single-copy and duplicated BUSCO scores were 94.1% and 4.9%, respectively. LAI scores were 21.0 and 20.7 for TalH_r1.0 and TalF_r1.0, respectively.

### Gene prediction and repeat sequence analysis

A total of 48,862 and 42,966 potential protein-coding sequences were identified based on *ab initio* prediction using Helixer in TalH_r1.0 and TalF_r1.0, respectively. Of these potential protein-coding sequences, 39,945 and 39,585 genes were located on the 8 pseudomolecule sequences (Table 1). The complete BUSCO score of TalH_r1.0 was 98.9%, and the single-copy and duplicate BUSCO scores were 93.4% and 5.5%, respectively. The BUSCO score of TalF_r1.0 was 99.1%, and the single-copy and duplicate BUSCO scores were 93.2% and 5.9%, respectively.

The accuracy of the predicted genes was assessed using Iso-Seq in 4 tissues: leaves, flowers, stems, and roots. A total of 1.52 million reads were clustered into 71k (leaf), 104k (flower), 100k (stem), and 9k (root) full-length transcripts, and the transcripts were further clustered into 166k unique transcripts. Similarly, Fahl exhibited full-length transcript counts of 77k (leaf), 79k (flower), 91k (stem), and 5k (root), totalling 150k unique transcripts. The complete BUSCO score of the unique transcripts was 94.6% for Helaly and 92.6% for Fahl. The predicted gene models with Helixer matched 145,410 (97.2%) and 135,440 (97.6%) of the unique transcripts for Helaly and Fahl, respectively, indicating that the accuracy of the predicted genes was well supported by the transcriptome data.

Repeat sequences occupied 354.2 Mb (64.67%) and 342.9 Mb (63.93%) in TalH_r1.0 and TalF_r1.0, respectively (Table S1). The dominant repetitive sequences were long terminal repeat (LTR) elements, constituting 27.79% of the sequences in Helaly and 29.55% in Fahl, followed by unclassified repeats, which constituted 14.64% of the sequences in Halely and 15.94% in Fahl. Retroelements and unclassified repeats were fewer in Helaly than in Fahl, whereas DNA transposons were more numerous in Helay than in Fahl (10.89% and 6.28%, respectively). The differences in repeat sequences between Helaly and Fahl included *hobo-Activator* elements, which were 15 Mb larger in Helaly (3 times more), primarily on chromosomes 2 (20 times more) and 4 (39 times more) (Fig. S3). MULE-MuDR elements were 5.5 Mb larger in Helaly (1.6 times more) across all chromosomes. In contrast, LTR elements were 6.3 Mb larger in Fahl (1.04 times more) across all chromosomes except chromosome 6.

### Ortholog analysis of the *T. alexandrinum* genes

**Fig. 4.**
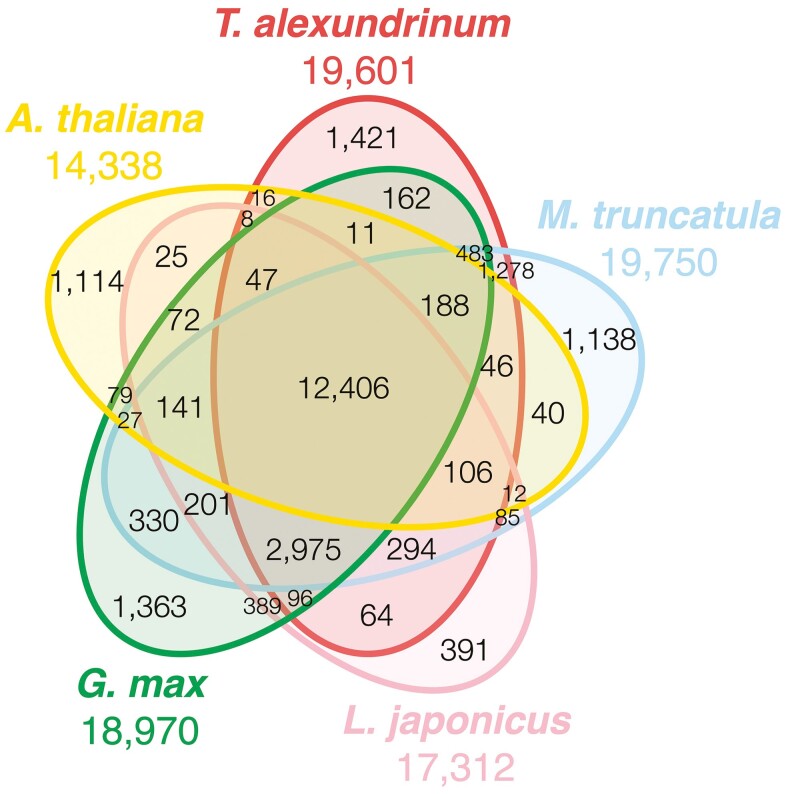
Venn diagram of the numbers of gene clusters in the 4 legume genomes and *A. thaliana*.

To evaluate the accuracy of gene prediction, we conducted an ortholog analysis focussing on orthogroup sharing and phylogenetic relationships, confirming evolutionary conservation, and identifying lineage-specific gene acquisition. The genes predicted in TalH_r1.0, which were used as representatives of *T. alexandrinum*, were clustered with those of *A. thaliana* and the 3 legume species *G. max*, *L. japonicus*, and *M. truncatula* (Figure 4). In total, 25,008 clusters were identified across the 5 species. Of these, 12,406 (49.6%) were shared among the 5 species, and 2,975 (11.9%) were shared among the 4 legume species. Species-specific clusters were identified, with 1,114 (4.45%) clusters found in *A. thaliana*, 1,363 (5.45%) in *G. max*, 391 (1.56%) in *L. japonicus*, 1,138 (4.55%) in *M. truncatula*, and 1,421 (5.68%) in *T. alexandrinum*. The number of clusters shared exclusively with *T. alexandrinum* was 16 (0.06%) for *A. thaliana*, 162 (0.65%) for *G. max*, 64 (0.26%) for *L. japonicus*, and 1,278 (5.11%) for *M. truncatula*.

### Population genomic analysis and GO enrichment analysis

To evaluate the genetic diversity between and within Egyptian clover cultivars, a genome-wide SNP analysis was performed. We obtained 958 million ddRAD-Seq reads from 144 Helaly and 894 million reads from 182 Fahl samples. After trimming and removing samples with reads of <30 million, the cleaned reads were mapped on the TalH_r1.0 assembly. Totals of 13,544 high-quality SNPs were detected in the genome.

**Fig. 5.**
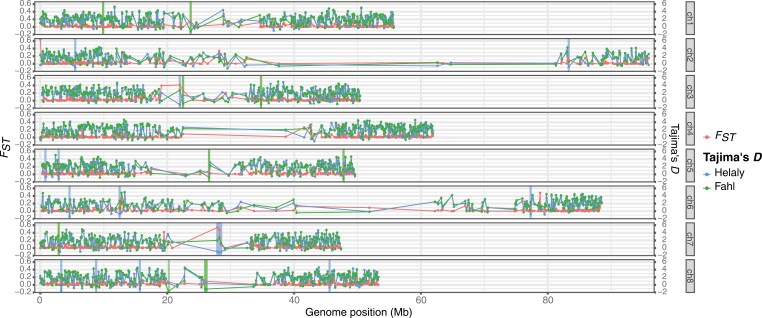
*F*_*ST*_ between cultivars and Tajima’s *D* within each cultivar along each chromosome with 100 kb sliding windows. Vertical lines represent genomic positions with significantly high *F*_*ST*_ and low Taima’s *D* in Helaly and Fahl, respectively.

Then, Tajima’s *D* was calculated for the whole genome using 100-kb sliding windows. The average Tajima’s *D* over the genome was 1.69 (–1.54 to 5.09) for Helaly and 1.85 (–1.72 to 5.34) for Fahl. The deviation of Tajima’s *D* values from 0 over the whole genomic region implies a particular population structure or dynamics rather than natural selection to a local genomic region. The positive values, which indicate the maintenance of higher genetic diversity than neutral over the genome, suggest that each cultivar is a composite population of subgroups. This conclusion is consistent with that Egyptian clover was bred using a mass selection method with pollinators, a common practice in forage breeding programs. Genome-wide *F*_*ST*_ values were also calculated to detect the genomic regions differentiated between the cultivars. In total, 246 genomic regions with significantly high *F*_*ST*_ were found throughout the genome (Fig. 5). Of the 246 genomic regions, 15 and 10 regions showed significantly low Tajima’s *D* values for Halely and Fahl, respectively. This result indicated that the high *F*_*ST*_ regions exhibited genetic differentiation between cultivars while the low Tajima’s *D* regions increased genetic uniformity probably due to purifying or directional selection.

We performed GO enrichment analysis on 142 genes in the 15 genomic regions for Halely to identify 10 significantly enriched GO terms (*P* < 0.01; Table S3) that included gene expression and DNA replication, metabolic and energy regulation, and development and formation. In parallel, GO enrichment analysis was performed on 82 genes within the 10 genomic regions for Fahl, revealing 7 significantly enriched GO terms (*P* < 0.01; Table S3) for metabolic and biosynthetic processes, environmental response, and development and formation. These results indicate that the characteristics specific to each cultivar might associated with at least these genomic regions and be explained by the GO terms enriched in the regions.

## Discussion

We constructed a near-T2T genome assembly consisting of 8 pseudomolecule sequences corresponding to the basic number of chromosomes in 2 Egyptian clover cultivars. The total assembly sizes of Helaly and Fahl were 547.7 Mb and 536.3 Mb, respectively. These sizes were larger than the estimated sizes for Helaly (486 Mb) and Fahl (474 Mb). High heterozygosity, repetitive sequences, and sequencing errors may have been responsible for the inconsistency between an assembly size and an estimated size based on *k*-mer frequency analysis.^53^ The underestimation observed in the present study may be due to the high heterozygosity and the abundance of repetitive sequences in the *T. alexandrinum* genome.

The assembly size for Helaly (547.7 Mb) was 11.4 Mb larger than that for Fahl (536.3 Mb), reflecting a size difference of 11.3 Mb in repeat sequences between the 2 cultivars: 354.2 Mb in Helaly and 342.9 Mb in Fahl. This difference in the size of the repeat sequences can be primarily attributed to the expansion of certain DNA transposons, *hobo*-*Activator* and MULE-MuDR, in the Helaly genome. MULE-MuDR expanded across entire chromosomes, while *hobo*-*Activator* locally expanded in chromosomes 2 and 4 (Supporting Figure S3). Both transposable elements are widespread among angiosperms and induce mutations that enhance genomic studies on maize.^54,55^ Furthermore, the transposable elements contribute to breeding by creating genetic diversity and enabling the selection of beneficial mutations.^56^

Improving the accuracy of gap sequences and the direction of small contigs is essential to produce more reliable genome assemblies. To achieve the near-T2T genome assemblies, we developed a novel scaffolding strategy, GAP-Seq, in which undetermined sequences in gap regions were targeted using the adaptive sampling method (ONT). Using the GAP-Seq approach, 61 contigs were connected in Helaly, improving the N50 length from 14.6 Mb to 21.3 Mb (~1.5 times). In Fahl, 79 contigs were connected, with the N50 length improving from 8.56 Mb to 22.1 Mb (~3 times). The contiguity of Fahl sequences was improved rather than that of Helaly. In the GAP-Seq with adaptive sampling, the target regions were simply set at the contig ends in Helaly but at the unique sequences of the contig ends in Fahl. The avoidance of redundant sequences as targets might contribute to the long scaffoldings. This method has an advantage over the practical target enrichment technologies that require molecular experiments using PCR or probes because the adaptive sampling method can computationally enrich the targets on the flow cell of the ONT sequencer without any molecular work. PacBio HiFi, together with GAP-Seq based on ONT adaptive sampling can be used for T2T genome assembly in other plant species.

Egyptian clover cultivars are not pure lines because of their allogamy, to avoid inbreeding depression. As reported in many previous studies,^57,58^ cultivar-specific characteristics can be identified using the population genomic analysis to examine genetic variations within a cultivar. In this study, we identified 15 and 10 genomic regions that were significantly differentiated between the 2 cultivars and increased genetic uniformity in each cultivar. The GO terms enriched in these regions included metabolic and biosynthetic processes, which may indicate the environment adaptability of each cultivar, as well as development and formation, which may explain differences in productivity between multi- and single-cut (Table S3). Differentiated loci may confer desirable traits during breeding. The identification of genetic loci for agriculturally important traits can facilitate Egyptian clover breeding programs with marker-assisted selection through genome-wide association studies and quantitative trait loci mapping.

In this study, we assembled the genome sequences of 2 Egyptian clover cultivars to reveal that the genome size and structure differed between the 2 cultivars. These data suggest that specific genomic sequences are present in cultivars and individuals. Therefore, high-quality genome sequences across cultivars and individuals, known as pan-genomics, are required to understand the genetic variations in the cultivars and individuals of a species.

## Supplementary Material

dsae036_suppl_Supplementary_Figure_S1

dsae036_suppl_Supplementary_Figure_S2

dsae036_suppl_Supplementary_Figure_S3

dsae036_suppl_Supplementary_Tables

dsae036_suppl_Supplementary_Material

## Data Availability

Raw sequencing reads and assemblies were deposited in the DNA Data Bank of Japan (DDBJ) under the accession number PRJDB818306. Genomic information is available from Plant GARDEN (https://plantgarden.jp/).
