# Supplementary figures and images for: Near-complete telomere-to-telomere de novo genome assembly in Egyptian clover (Trifolium alexandrinum)
Source: DNA Res. 2024 Dec 18;32(1):dsae036. doi: 10.1093/dnares/dsae036 (PMC11747361; doi:10.1093/dnares/dsae036)

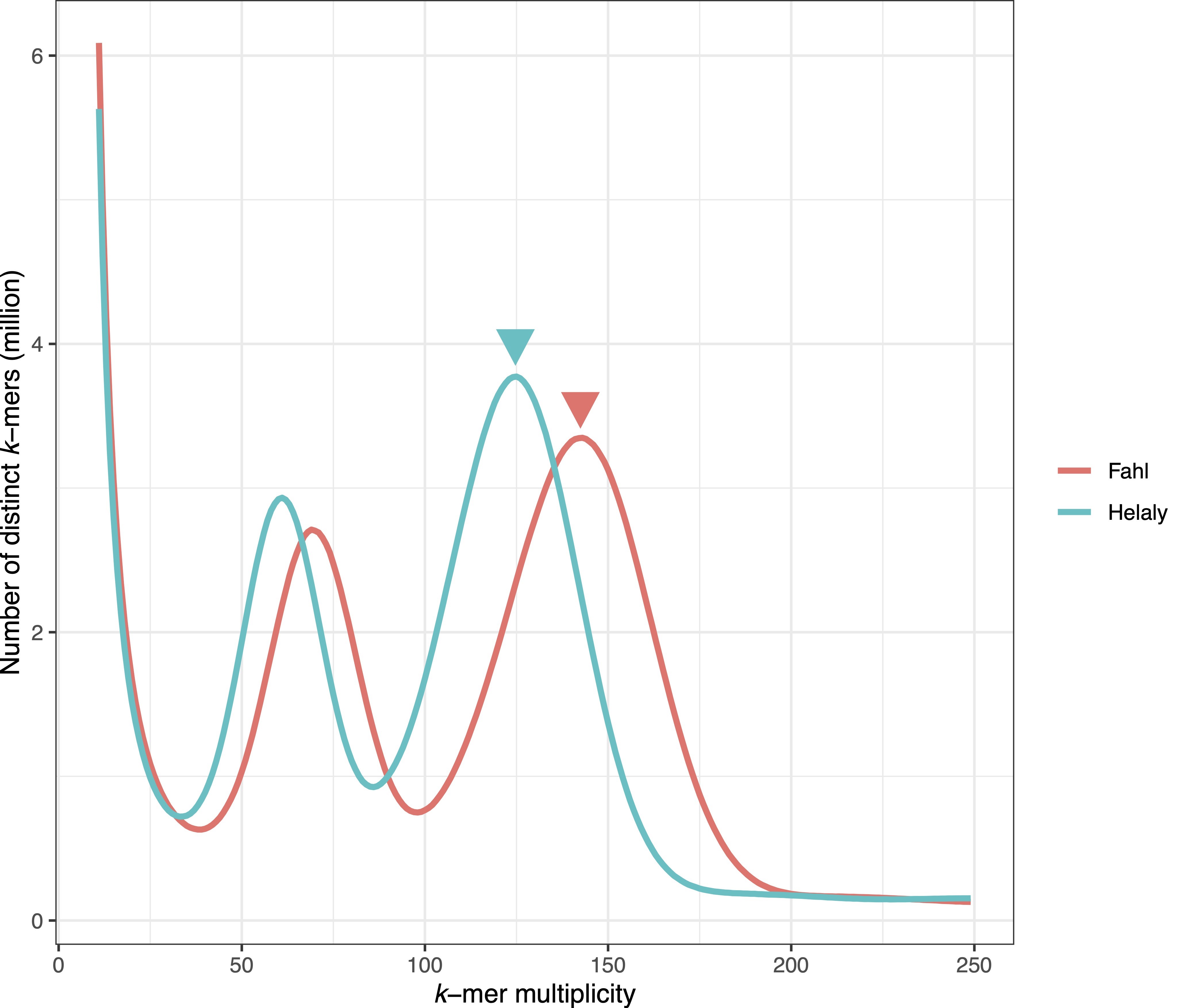

Supplement: dsae036_suppl_Supplementary_Figure_S1 [file dsae036_suppl_supplementary_figure_s1.jpeg]

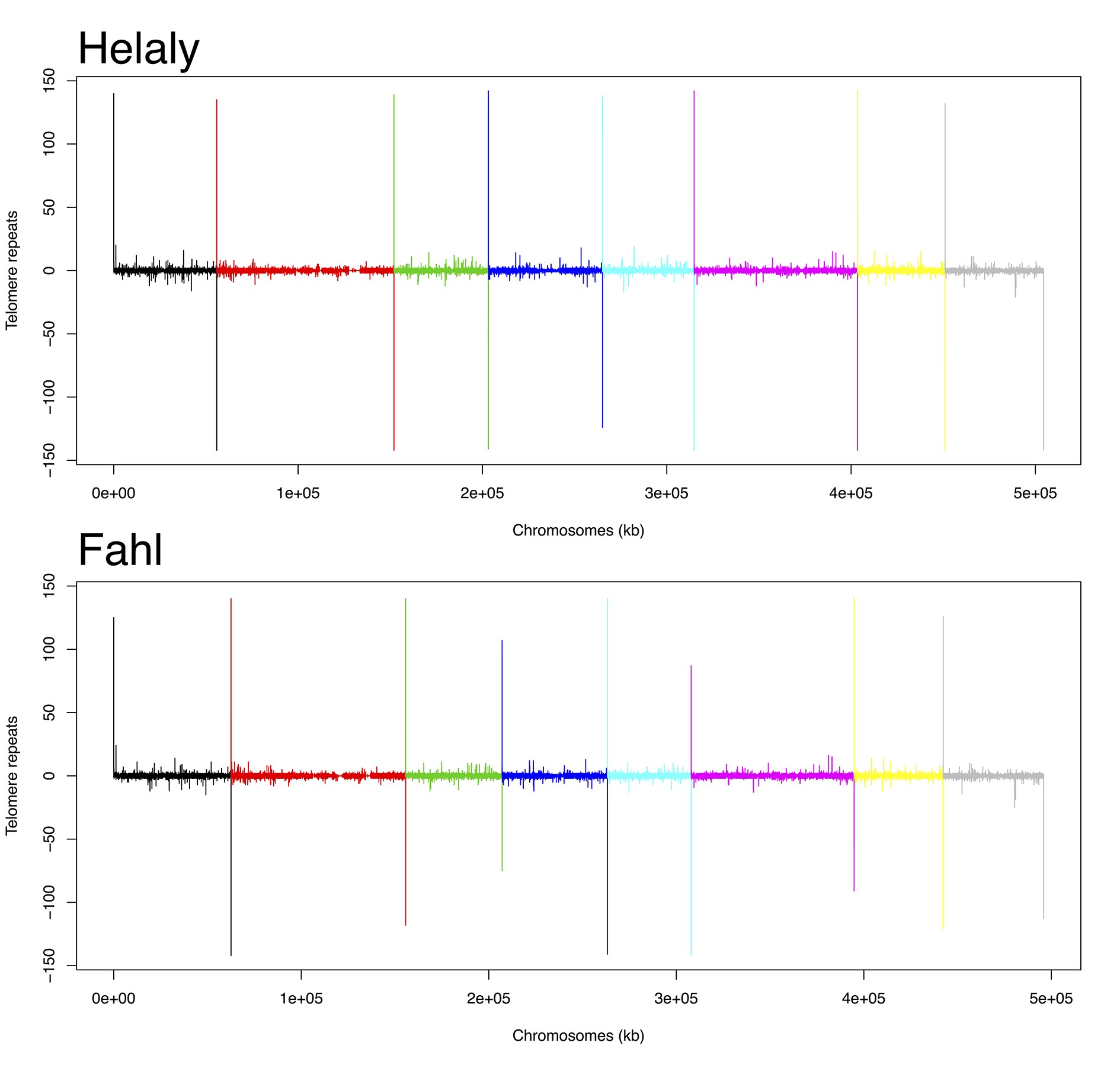

Supplement: dsae036_suppl_Supplementary_Figure_S2 [file dsae036_suppl_supplementary_figure_s2.jpeg]

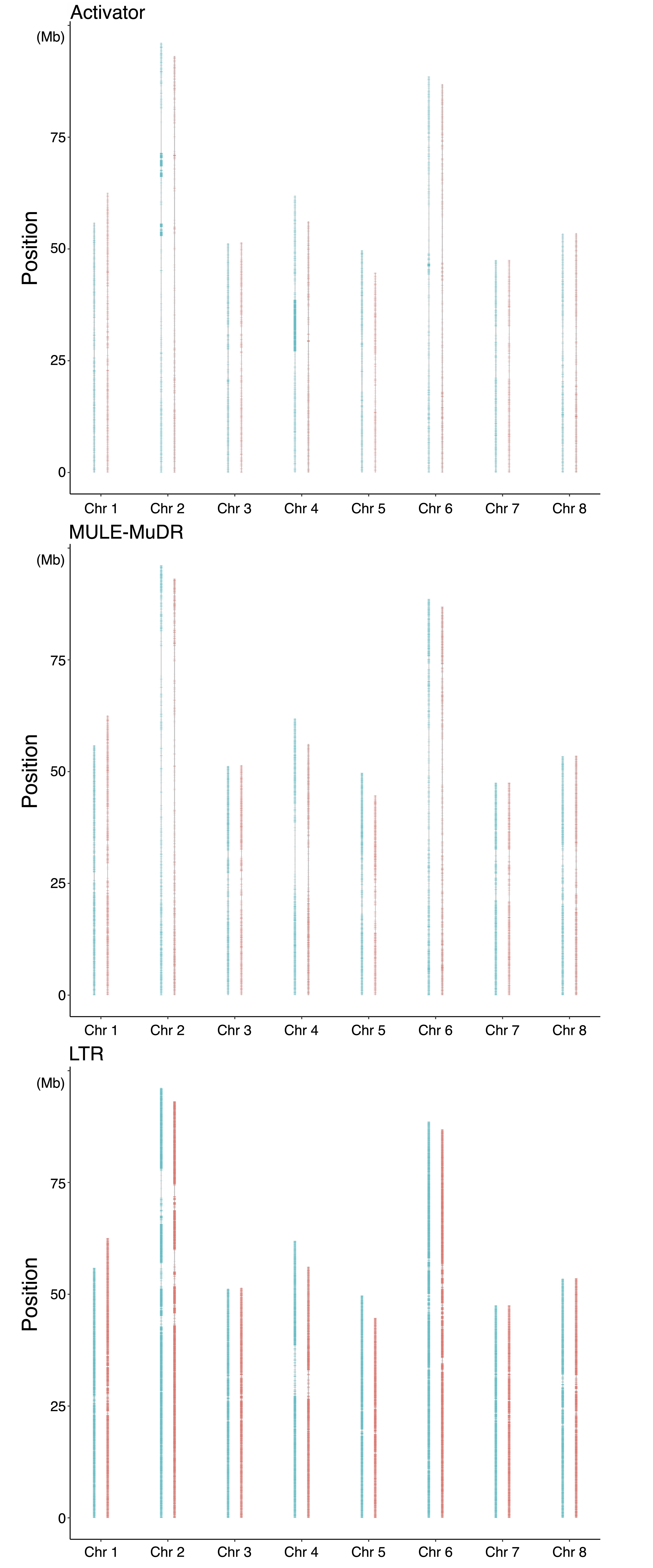

Supplement: dsae036_suppl_Supplementary_Figure_S3 [file dsae036_suppl_supplementary_figure_s3.jpeg]
