# Supplementary material for: Near-complete telomere-to-telomere de novo genome assembly in Egyptian clover (Trifolium alexandrinum)
Source: DNA Res. 2024 Dec 18;32(1):dsae036. doi: 10.1093/dnares/dsae036 (PMC11747361; doi:10.1093/dnares/dsae036)
Supplement: dsae036_suppl_Supplementary_Material [file dsae036_suppl_supplementary_material.pdf]

Supplementary Information

Near-complete telomere-to-telomere *de novo* genome assembly in Egyptian clover (*Trifolium alexandrinum*)

Mitsuhiko P. Sato[1], Ramadan A. Arafa[2], Mohamed Rakha[3], Amero A. Emeran[3], Sachiko Isobe[1], and Kenta Shirasawa[1*]

[1] Kazusa DNA Research Institute, Chiba, 292-0818, Japan

[2] Plant Pathology Research Institute, Agricultural Research Center, Giza, 12619, Egypt

[3] Faculty of Agriculture, Kafrelsheikh University, Kafr El-Sheikh 33516, Egypt

[*]To whom correspondence should be addressed:
Kenta Shirasawa
Tel. +81 438 52 3935
Email: shirasaw@kazusa.or.jp

Supporting Figure S1. Estimation of the genome size of *T. alexandrinum*, based on *k*-mer analysis ($k = 21$) with given multiplicity values. Triangles indicate the peak used by estimation.

Supporting Figure S2. The distribution of telomere repeats along eight chromosomes for Helaly and Fahl. Colors indicate each chromosome.

Supporting Figure S3. Genomic positions of the repeat sequences that differ in the copy number between the two cultivars. Blue indicates Helaly and red indicates Fahl.

Supporting Table S1. Repetitive sequences in TalH_r1.0 and TalF_r1.0.

Supporting Table S2. Number of reads and ddRAD-Seq mapping rates.

Supporting Table S3. Enriched gene ontology list of genomic regions genetically differentiated between two cultivars.
